# Epidemic Infantile Paralysis

**Published:** 1897-07-17

**Authors:** 


### EPIDEMIC INFANTILE PARALYSIS.

On April 3rd, we reported an interesting paper by Dr. W. Pasteur, in which he related a series of cases of infantile paralysis, or at any rate of cases presenting great analogies with that condition, which had occurred among children of the same family. In commenting on this paper, Dr. Buzzard remarked that this was the first occasion in which the infective character of the disease causing infantile paralysis had been brought before an English medical society, and added that he felt very sure that infantile paralysis arose from some toxic influence, and that this was not limited to the anterior cornea of the spinal cord. As bearing upon this point, it is interesting to record a remarkable epidemic of 14 cases of acute anterior poliomyelitis, details of which are given by Mr. C. A. Altmann, of Lincoln, South Australia, in the *Australasian Medical Gazette.* All the cases occurred in the same district during March and April, 1895, since when the author had met with no fresh cases, while the last case previous to the epidemic was an isolated one in 1887. In two instances two members of the same family were attacked in rapid succession. All the cases seem to have been in good health up to the time of attack. The weather was hot and dry at the time of the outbreak. All the patients except one were over three years old, and, in fact, many of them were much above the age at which the disease most commonly occurs. The ages, in years, were as follows: 13, 10, 16, 26, 13, 3, 4, 1, 7, 4, 5, 4, 8, 5.

From a perusal of the records we can have no doubt that this was a genuine epidemic of this strange disorder. In those observed at the time of onset there were generally raised temperature, pain, and tenderness, with paralysis and loss of faradic irritability, and ultimately wasting of certain muscles. In some the leg, in others the arm was affected, and in one case the paralysis took the form of hemiplegia, as occurred in one of the cases reported by Dr. Pasteur. An interesting fact in regard to the epidemic was that it coincided with a general use of phosphorus in the district, for the purpose of destroying the rabbits which existed there in almost countless numbers. In January the Port Lincoln District Council issued instructions that during the months of March and April simultaneous efforts were to be made throughout the whole district for the destruction of the rabbits. The general method adopted for this purpose consisted in laying phosphorised pollard, and about 200 lbs. of white phosphorus were sold in the district for the purpose. In all but two of the houses where the epidemic occurred phosphorus was being used, and during the same months several cases of mild phosphorus poisoning were observed in adults. This may have been a mere coincidence, but it is interesting. So far as treatment was concerned the results were disappointing, as indeed is always the case.

